# Integrin α5β1 is necessary for regulation of radial migration of cortical neurons during mouse brain development

**DOI:** 10.1111/j.1460-9568.2009.07072.x

**Published:** 2010-02

**Authors:** Giovanni Marchetti, Sarah Escuin, Arjan Van Der Flier, Adèle De Arcangelis, Richard O Hynes, Elisabeth Georges-Labouesse

**Affiliations:** 1 Institut de Génétique et de Biologie Moléculaire et Cellulaire, Department of Cell Biology and DevelopmentCNRS UMR7104, Inserm U964, Université de Strasbourg, 67404 Illkirch, France; 2 Howard Hughes Medical Institute, Koch Institute for Integrative Biology, Massachusetts Institute of Technology CambridgeMA, USA

**Keywords:** cell adhesion, cerebral cortex, embryo, integrin, motility

## Abstract

During cerebral cortex development, post-mitotic neurons interact with radial glial fibers and the extracellular environment to migrate away from the ventricular region and form a correct laminar structure. Integrin receptors are major mediators of cell–cell and cell–extracellular matrix interactions. Several integrin heterodimers are present during formation of the cortical layers. The α5β1 receptor is expressed in the neural progenitors of the ventricular zone during cerebral cortex formation. Using *in utero* electroporation to introduce short hairpin RNAs in the brain at embryonic day 15.5, we were able to inhibit acutely the expression of α5 integrin in the developing cortex. The knockdown of α5 integrin expression level in neural precursors resulted in an inhibition of radial migration, without perturbing the glial scaffold. Moreover, the same inhibitory effect on neuronal migration was observed after electroporation of a Cre recombinase expression plasmid into the neural progenitors of conditional knockout mice for α5 integrin. In both types of experiments, the electroporated cells expressing reduced levels of α5 integrin accumulated in the premigratory region with an abnormal morphology. At postnatal day 2, ectopic neurons were observed in cortical layer V, while a deficit of neurons was observed in cortical layer II–IV. We show that these neurons do not express a layer V-specific marker, suggesting that they have not undergone premature differentiation. Overall, these results indicate that α5β1 integrin functions in the regulation of neural morphology and migration during cortical development, playing a role in cortical lamination.

## Introduction

In the developing cerebral cortex, a large number of neurons are generated in the ventricular (VZ) and sub-ventricular zone (SVZ) of the dorsal telencephalon and migrate radially along the glial fibers, to reach their final position in the cortical plate (CP) (Nadarajah *et al.*, 2003). The correct formation of the cortical layers requires that migrating neurons, from the premigratory zone to the CP, undergo a morphological transition from a multipolar to a bipolar shape (Kriegstein & Noctor, 2004; Noctor *et al.*, 2004; LoTurco & Bai, 2006). Adhesive interactions between the extracellular matrix (ECM), neurons and radial glia are likely to play important roles during this process of neuronal migration (Schmid & Anton, 2003; Sarkisian *et al.*, 2008). Major cell surface receptors for ECM are the integrins, which are heterodimers constituted by α and β subunits that bind specific ECM ligands [e.g. fibronectin (FN), laminin (LN) or collagen] (Hynes, 2002). Previous studies have suggested that α3 and αv integrin subunits may be important for interactions between migrating neurons and radial glia, whereas the integrin subunits α6 and β1 regulate anchorage of glial endfeet and meningeal basement membrane remodeling (Georges-Labouesse *et al.*, 1998; Anton *et al.*, 1999; Graus-Porta *et al.*, 2001; Belvindrah *et al.*, 2007). Regarding ECM ligands, the role of laminin in the organization of the mouse cortex has been demonstrated (Miner *et al.*, 1998; Halfter *et al.*, 2002; Chen *et al.*, 2009). And some evidence points to a role for FN as a potential ligand for guiding radial neuronal migration and regulating the interactions between neurons and radial glia. In particular, high levels of FN mRNA were found in the VZ during early corticogenesis and in the CP at later stages (Sheppard *et al.*, 1995). Stettler & Galileo (2004) observed that, in the chick optic tectum, FN was produced by radial glial fibers and was aligned along their surfaces during neuronal migration. Early embryonic lethality of FN-null embryos has so far precluded phenotypical studies in the developing nervous system. In addition, embryos lacking the α5 integrin subunit, which forms the α5β1 major FN receptor, also die at embryonic day (E)10.5 before cerebral cortex development (Yang *et al.*, 1993; Goh *et al.*, 1997). Recently, α5β1 has been implicated in regulating morphology of dendritic spines and formation of synapses in neurons (Webb *et al.*, 2007), as well as in mediating neurite outgrowth after injury (Gardiner *et al.*, 2007).

We developed two strategies to overcome the early embryonic lethality and analyse the potential roles of α5β1 integrin in neuronal migration during brain development. First, we introduced short hairpin RNA (shRNA) against α5 integrin by *in utero* electroporation in embryonic brains to inhibit its expression in VZ and SVZ cells. Second, starting from α5 integrin floxed mice (A. van der Flier *et al.*, unpublished data), we introduced a Cre plasmid also by *in utero* electroporation to delete the *α5* gene in VZ/SVZ cells (Calegari *et al.*, 2004; Chen *et al.*, 2008). Using the two approaches, we found that inhibition of α5β1 expression in VZ and SVZ leads to defects in neuronal migration.

## Materials and methods

### Plasmids

α5 integrin target sequences were designed via the GenScript shRNA design tool and are as follows: shRNA-1 (R1), 5′-CCTGCTACCTCTCCACAGAAA-3′; shRNA-4 (R4), 5′-GCAGATCTCGGAGTCCTATTA-3′; shRNA-7 (R7), 5′-CTGCCTCAATGCCTCTGGAAA-3′; shRNA-9 (R9), 5′-ACTTTCAGATCCTCAGCAAGA-3′; control scrambled shRNA (Ctr), 5′-CACAATATCTGCCCCGATCCA-3′. shRNA constructs were generated in the pRNAT-U6.1/Neo vector (GenScript, Paris, France), which allows expression of a coral green fluorescent protein (GFP) marker with the shRNA of interest. Full-length mouse α5 integrin (German Science Center for Genome Research RZPD, Berlin, Germany) and α6 integrin were cloned into pcDNA3.1 (Invitrogen, Cergy-Pontoise, France). For Cre-recombination experiments, we used the pCIG2 plasmid (kind gift of F. Polleux, University of North Carolina), encoding enhanced GFP (EGFP), and pxCANCre expressing Cre recombinase under the control of the CAG promoter (obtained from DNA Bank, Tsukuba Life Science Center, RIKEN, Japan).

### Antibodies

Primary antibodies used in this study were against integrin α5 (rabbit, 1 : 500, Chemicon, Temecula, CA, USA; rat, 1 : 50, Pharmingen, Le Pont de Claix, France), integrin α6 (rat, 1 : 50, Pharmingen), bromodeoxyuridine (BrdU) (mouse, 1 : 100, Boehringer Mannheim), cleaved caspase 3 (Casp-3) (rabbit, 1 : 500, Cell Signalling, Saint Quentin en Yvelines, France), GFP (rabbit, 1 : 1000, Molecular Probes, Leiden, The Netherlands), glyceraldehyde 3-phosphate dehydrogenase (GAPDH) (mouse, 1 : 300, Chemicon), nestin/Rat 401 (mouse, 1 : 10, Developmental Studies Hybridoma Bank, Iowa City, IA, USA), chicken ovalbumin upstream promoter transcription factor-interacting protein 2 (Ctip2) (rat, 1 : 500, Abcam, Cambridge, UK), CCAAT-displacement protein (Cdp) (rabbit, 1 : 50, Santa Cruz Biotechnology) and integrin β1 (rat, 1 : 100, Chemicon). Secondary antibodies used for immunochemistry or in Western blotting analyses were Alexa fluor 488 goat anti-rabbit IgG (Molecular Probes), Alexa fluor 594 goat anti-mouse IgG (Molecular Probes), horseradish peroxidase (HRP) goat anti-rabbit, anti-mouse and anti-rat (Jackson ImmunoResearch Laboratories, West Grove, PA, USA).

### Cell lines and Western blot analysis

To evaluate the efficiency of shRNA constructs, HeLa cells were transiently transfected using Effectene reagent (Qiagen, Les Ulis, France). Two days after transfection, cells were lysed in RIPA buffer [150 mm NaCl, 10 mm Tris–HCl (pH 7.4), 0.1% sodium dodecyl sulfate (SDS), 1% Triton X-100, 1% sodium deoxycholate, 5 mm EDTA and a protease inhibitor cocktail (Roche, Mannheim, Germany)]. The lysates were cleared by centrifugation at 17 000 ***g*** for 15 min at 4°C. The proteins were separated by 8% SDS-PAGE and transferred to nitrocellulose membranes (Schleicher and Schuell, Dassel, Germany) for Western blotting.

### Mice

All animal experiments were approved by the regional ethics committee for animal experimentation (CREMEAS) and in strict accordance with French regulations.

shRNA plasmid injections were performed in CD1 wild-type mice. For the production of integrin α5 floxed mice, a previously isolated genomic clone (Yang *et al.*, 1993) was used to clone the α5 integrin conditional targeting construct. The targeting vector contained the following elements: a CM1-TK cassette, followed by 2-kb 5′-genomic arm, an Frt-flanked PGF-neo cassette, the 1.5-kb genomic sequence containing the exon 1 (255 bp) was flanked by loxP sites, followed by a 4-kb 3′-genomic arm. R1 embryonic stem (ES) cells were electroporated, selected and screened for correct recombination and single integration by Southern blotting analysis. Subsequently, the PGK-neo cassette was removed *in vitro* by transient expression of Flip recombinase. Two karyotyped and correctly targeted ES clones gave germline transmission and gave identical experimental results. Cre-mediated excision of exon 1 has been confirmed by PCR and Southern blotting in α5-floxed mice crossed to various transgenic Cre-strains as well as in derived cells. Loss of α5 protein after Cre-mediated recombination in several transgenic Cre-expressing strains could be detected by fluorescence-activated cell sorting, immunoblots and immunohistochemistry (A. van der Flier *et al.*, unpublished data).

### In utero electroporation

shRNA constructs or the pxCANCre plasmid were transfected by *in utero* electroporation as previously described (Langevin *et al.*, 2007; S. Escuin and E. Georges-Labouesse, unpublished data). Briefly, pregnant CD1 or *α5*^*f/f*^ mice and control mice were anesthetized by isoflurane inhalation and 1–3 μL plasmids (3 μg/μL) with 1% Fast Green was microinjected into the lateral ventricles of each embryonic brain at E15.5 using pulled glass capillaries. Electroporation was performed by delivering five 50-V pulses of 50 ms each at 950-ms intervals using the ECM electroporator (BTX, Holliston, MA, USA). The uterine horns were then placed back in the abdominal cavity to allow the embryos to continue development. The transfected brains were analyzed 2, 3 or 6 days after electroporation.

### BrdU labeling

For proliferation studies, pregnant mice were injected intraperitoneally with 50 mg/kg body weight of BrdU (Boehringer Mannheim) 48 h after *in utero* electroporation. The electroporated brains were harvested 2 h after BrdU injection. In all experiments, mice were killed by cervical dislocation and anesthetized by isoflurone inhalation.

### In situ hybridization

Frozen sections were processed for *in situ* hybridization with ^35^S-labeled RNA probes as described in Georges-Labouesse *et al.* (1998). Fragments used as probes were a 5′ fragment of α5 integrin cDNA (860 nt) or a 1-kb β1 cDNA (gift from R. Fässler, Max Planck Institute, Martinsried, Germany).

### X-Gal staining

Frozen sections were washed with phosphate-buffered saline (PBS) and stained with 1 mg/mL X-Gal (Promega, Paris, France) in PBS containing 5 mm K_3_Fe(CN)_6_, 5 mm K_4_Fe(CN)_6_ and 2 mm MgCl_2_ overnight at 37°C. Sections were then rinsed with PBS and mounted in Eukitt (EMS, Harrisburg, PA, USA).

### Immunohistochemistry and confocal microscopy

Embryonic brains were fixed in 4% paraformaldehyde in PBS, cryopreserved in 10% then in 20% sucrose/PBS and 10-μm coronal sections were cut with a Leica cryostat (Wetzlar, Germany). Sections were permeabilized in PBS/0.1% Tween 20 and stained with primary antibodies overnight at 4°C. After washing, slides were incubated for 1 h at room temperature with secondary antibodies. Images were acquired on a Macroconfocal LSI developed by the imaging center of IGBMC (Leica Microsystems), a laser scanning confocal microscope (Leica) with a 40×/1.25 or 63×/1.40 oil-immersion objective, or a Leica DMRBP fluorescence microscope.

### Statistical and quantification analyses

The dorsal–lateral region of the cerebral cortex was analysed for all electroporation experiments. To determine significant changes relative to control electroporations, in general, at least three independent electroporated brains were processed for each DNA condition. For each sample, three or four adjacent sections (300 cells) were analysed using a Macroconfocal LSI (Leica). Different subregions of the cerebral cortex were identified and visualized based on cell density using 4′,6-diamidino-2-phenylindole (DAPI) staining (Molecular Probes) and GFP-positive cells were counted and assessed for their location. The fraction of GFP-positive cells from each brain compartment in the shRNA or Cre-electroporated brains were compared with the fraction of the equivalent compartment in the control conditions. Microsoft Excel software was used to analyse the data for statistical significance. Statistical analysis was performed using two-tailed Student’s *t*-test between control and experimental conditions. Results are indicated as mean ± SEM. The mean was calculated and set to 100%. *P* values below 0.02 were considered statistically significant and are indicated in each figure legend.

## Results

**FIG. 1:**
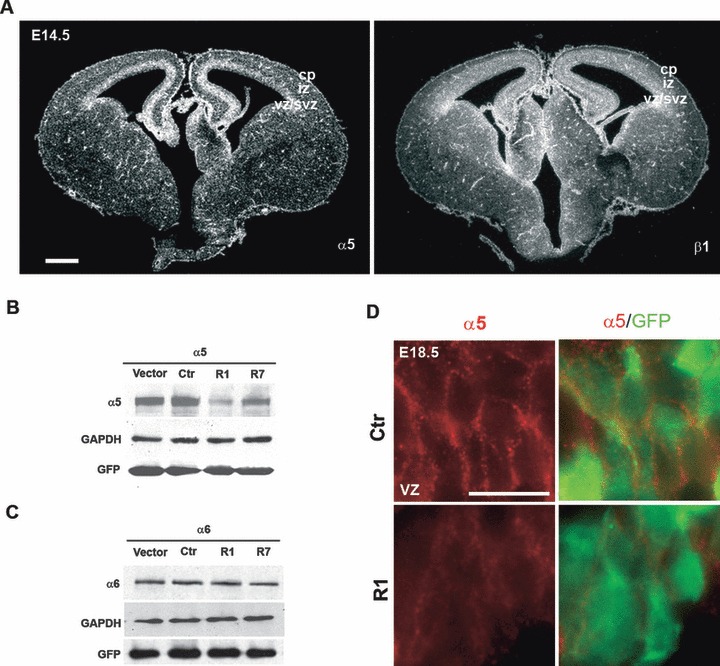
Analysis of the expression and knockdown efficiency of α5β1 integrin in the developing murine neocortex. (A) *In situ* hybridization of coronal brain sections at E14.5 revealed a signal in the VZ/SVZ for the α5 subunit; the signal for the β1 subunit was widespread throughout the laminar structure of the cerebral cortex and visible in the VZ and SVZ in some areas. Scale bar, 500 μm. (B, C) pcDNA3.1 vector containing an α5 (B) or α6 (C) integrin murine cDNA was cotransfected into HeLa cells with pRNAT-U6.1/Neo empty vector, the control construct (Ctr), or two different shRNA constructs targeting mouse α5 integrin (R1 and R7). Forty-eight hours after transfection, cells were lysed and were subjected to immunoblotting for α5 integrin or α6 integrin. GAPDH and GFP are, respectively, loading and transfection efficiency controls. α5-shRNA constructs were able to decrease α5 integrin expression by ∼80% (R1) and ∼40% (R7) but had no detectable effect on expression of the α6 integrin subunit. (D) High magnification of the VZ of E18.5 brain sections after electroporation with R1 α5-shRNA. Immunostaining for α5 integrin (red) and GFP (green) showed a reduction of endogenous α5 integrin expression in the cortical neuronal progenitors compared with the control brain. Scale bar, 50 μm. VZ/SVZ, ventricular zone/subventricular zone; IZ, intermediate zone; CP, cortical plate.

It has been previously reported that the α5 integrin subunit is expressed in the ventricular region of the mouse developing cerebral cortex (Yoshida *et al.*, 2003). To confirm and refine this localization, we performed *in situ* hybridization with α5 or β1 RNA probes on coronal sections of E14.5 mouse brains. In addition to a signal in blood capillaries, a strong signal for α5 integrin was detected in the ventricular zone, the region where neural cortical progenitors are located (Fig. 1A). The β1 expression domain was broader (VZ/SVZ and CP), reflecting the expression of other αβ1 integrins (Georges-Labouesse *et al.*, 1998). Staining experiments with antibodies at E14.5 confirmed the expression of α5 in neural progenitors and blood capillaries, but not in radial glial fibers or glial endfeet, while integrin α6 and β1 showed broader expression domains including the VZ and the CP and the nestin-positive radial glial fibers (Fig. S1).

To investigate the functional role of α5β1 integrin in neural progenitors, we used electroporation-mediated gene transfer to induce α5 integrin acute down-regulation by RNA interference. To reduce the expression of α5 integrin, four shRNA constructs were designed, against different regions of α5 integrin mRNA. BLAST (NCBI) searches with the shRNA sequences revealed no significant homology with other integrin genes or other genes. To produce shRNA, we used the pRNAT-U6/neo vector (GenScript), which allows the expression of shRNA and coral (c)GFP. This vector has been used successfully to knockdown the *Lis1* gene (Tsai *et al.*, 2005). Effective suppression of α5 integrin expression was tested by cotransfecting mouse α5 integrin cDNA with each shRNA construct into HeLa cells. By Western blot analysis of cells lysed 48 h after transfection, we observed that α5-shRNA 1 (R1) very efficiently decreased α5 integrin expression, while α5 shRNA 7 (R7) had an intermediate effect, when compared with α5 integrin protein levels in cells transfected with empty vector or control scrambled α5-shRNA construct (Ctr) (Fig. 1B). Cotransfection with α5-shRNA 4 and 9 (called R4 and R9) did not reduce α5 integrin expression, such that α5 integrin protein levels were similar to transfection with empty vector or Ctr construct (data not shown). To test the specificity of the R1 and R7 constructs for the α5 integrin chain, we transiently cotransfected HeLa cells with mouse α6 integrin cDNA and R1 or R7 α5-shRNA constructs. Transfection of α5-shRNA constructs did not affect the expression of the α6 integrin chain (Fig. 1C).

Constructs containing α5-shRNAs (R1 and R7), or Ctr shRNA, or empty vector were introduced into neural progenitor cells by electroporation in the mouse neocortex at E15.5. This stage allowed us to target the waves of later-born post-mitotic neurons which will ultimately form the cortical layers II–IV at postnatal stages. Embryonic brains were harvested 3 days after electroporation at E18.5. First, we confirmed that the R1 construct was able to knockdown endogenous α5 integrin protein *in vivo* in the developing neocortex by performing staining for α5 integrin on coronal sections from R1 or control shRNA electroporated brains. In areas where GFP-positive cells were located, a decrease in the level of integrin α5 signal was observed in those brains electroporated with R1 but not in control brains (Fig. 1D).

It has been shown previously that αβ1 receptors play a role in the regulation of neural progenitor cell proliferation, survival and migration in neurosphere cultures derived from postnatal day (P)1 mice (Leone *et al.*, 2005). To determine whether the down-regulation of α5 integrin perturbs cell proliferation of neural precursors at embryonic stages, BrdU incorporation experiments were performed. For this, BrdU was injected into experimental pregnant females at E17.5, 2 days after electroporation, and embryonic brains were collected 2 h after BrdU injection. After double immunostaining with antibodies against BrdU and GFP, we counted the number of BrdU and GFP double positive cells and compared this with the total number of GFP-positive cells in the VZ and SVZ of control (Ctr, *n* = 3) or R1 (*n* = 3) injected embryos. As shown in Fig. S2, A, there was no significant difference (*P* = 0.45) in the fraction of BrdU-positive cells between Ctr (11.5 ± 0.6%) and R1 (11.4 ± 1.5%), suggesting no impairment in proliferation rate. The same result was obtained with a phospho-histone H3 (pH3) staining at E18.5 (data not shown). A reduction of integrin could also affect cell survival. To assess cell death, activated caspase-3 labeling was performed on electroporated brain sections. At this stage, very few cells were positive for caspase-3 in both conditions. Thus, α5 integrin knockdown did not have significant effects on apoptotic cell death of cortical progenitors (Fig. S2B).

**FIG. 2:**
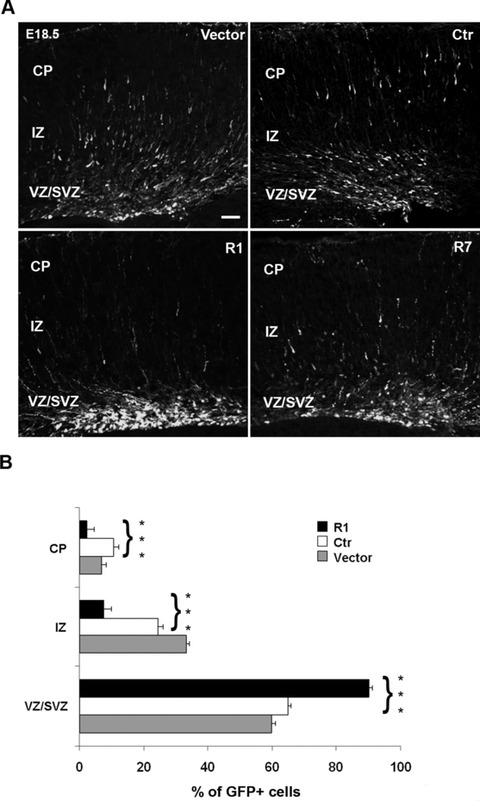
Effect of α5 integrin knockdown in developing murine cerebral cortex. (A) Representative coronal sections of embryonic murine neocortex 3 days (E18.5) following electroporation of empty vector, Ctr, R1 and R7 α5 shRNAs together with coral GFP. Transfection of R1 and R7 shRNAs impaired radial migration. Scale bar, 100 μm. (B) Quantitative analyses of the distribution of GFP-positive cells in the various cortical layers. The cortical wall was subdivided, and numbers of GFP-positive cells were counted in each layer and expressed as a percentage of the total. Statistical differences were seen for each layer, comparing the distribution of GFP-positive cells between Ctr and R1 α5-shRNA. Asterisks indicate significant differences between the groups (***P < 0.001, using Student’s *t*-test).

To analyse further the defects induced by the knockdown of α5, we investigated the distribution of electroporated cells in E18.5 brains. Electroporation of empty vector or control shRNA construct did not affect the distribution of electroporated cells which were found in the VZ/SVZ, intermediate zone (IZ) and CP. In contrast, in coronal sections of brains electroporated with the R1 construct, the majority of GFP-positive cells were located in the VZ/SVZ with a limited number of cells in the IZ and CP (Fig. 2A). Electroporation of the R7 construct induced milder alterations as compared with the effects of the R1 construct, but still resulted in an abnormal distribution of GFP-positive cells (Fig. 2A).

To quantify the defects, we counted GFP-positive cells in the VZ/SVZ, IZ and CP regions of brains electroporated with the R1 construct (*n* = 6), with empty vector (*n* = 4) and with control construct (*n* = 4). In control brains, transfected with either the empty vector or the Ctr construct, we observed the expected distribution of GFP-positive cells in the VZ/SVZ (empty vector: 59.8 ± 1.0%; Ctr: 64.9 ± 2.0%), in the IZ (empty vector: 33.2 ± 0.8%; Ctr: 24.4 ± 1.4%) and in the CP (empty vector: 6.9 ± 1.2%; Ctr: 10.6 ± 2.0%) (Fig. 2B). In contrast, in brains that were electroporated with the α5-shRNA R1 construct, the majority of transfected cells were still located in the VZ/SVZ (90.0 ± 2.0%), with a small subset in the IZ (7.6 ± 1.4%) and CP (2.2 ± 1.0%) (Fig. 2B). These differences in the distribution of GFP-positive cells between the R1 and Ctr electroporated brains were statistically significant for each region (*P* < 0.001). An increase in the VZ/SVZ and a marked decrease in upper layers, without changes in proliferation or cell death, suggest that it is the migration process which is disturbed by the down-regulation of α5 integrin in neural progenitor cells.

**FIG. 3:**
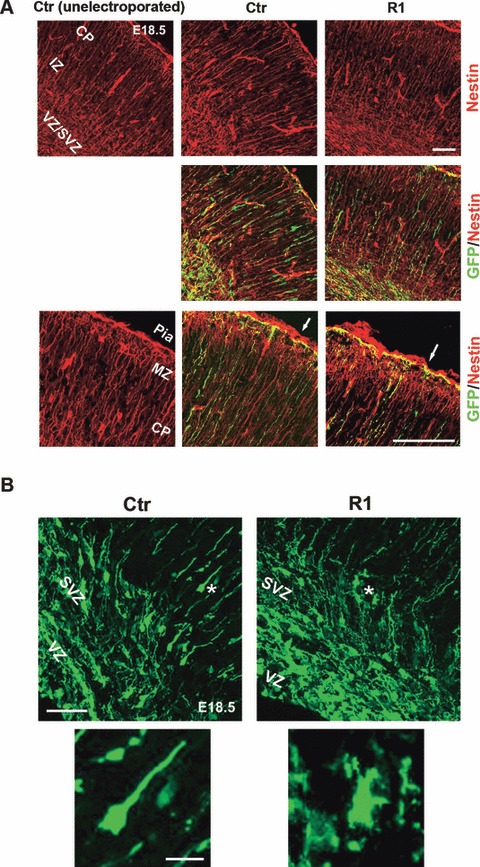
Influence of α5-shRNA on radial glial scaffold and neuronal morphology. (A) Coronal sections of unelectroporated hemisphere and of brains electroporated at E15.5 with Ctr and R1 α5-shRNAs were stained with anti-Nestin (red) and GFP (green) antibodies. GFP-positive radial fibers transfected with R1 α5-shRNA appeared normal as compared with unelectroporated and Ctr α5-shRNA-transfected fibers. Scale bar, 100 μm. Bottom panels are magnified views of the MZ. The white arrows indicate basal endfeet of radial fibers attached to the pia. Transfection of R1 α5-shRNA does not seem to disturb formation of glial endfeet. MZ, marginal zone. Scale bar, 50 μm. (B) Morphology of VZ/SVZ neurons transfected with Ctr or R1 α5 shRNAs. The electroporated cells with Ctr shRNA showed a normal bipolar morphology in the premigratory region (white asterisk, high magnification). In contrast, cells expressing shRNAs against α5 integrin are arrested within the VZ/SVZ with a multipolar shape (white asterisk, high magnification). Scale bars: top, 50 μm; bottom, 10 μm.

Previous studies reported that deletion of the α6 or β1 integrin subunits in the brain leads to defects in cortical laminar organization, associated with perturbations in the anchorage of radial glial fibers at the meningeal basement membrane (Georges-Labouesse *et al.*, 1998; Graus-Porta *et al.*, 2001). To test whether the knockdown of α5 integrin in progenitors of the VZ at E15.5 perturbed the morphology of radial glial fibers or the formation of glial endfeet, coronal sections of brains electroporated with R1 and control constructs were immunostained with an anti-nestin antibody. GFP-nestin-positive radial glial fibers, containing the R1 construct, did not show any major morphological differences or glial endfeet defects compared with GFP-nestin-positive radial glia expressing a control construct (Fig. 3A). Thus, the reduction of integrin α5β1 by electroporation at E15.5 did not induce major abnormalities in aspect or attachment of radial glial fibers.

It has been well documented that migration of cortical neurons is a complex process that includes radial glia-dependent and glia-independent steps (LoTurco & Bai, 2006). Recently, *in utero* electroporation of GFP plasmids followed by live imaging has allowed visualization of a population of multipolar cortical neurons which seem to be transient intermediates during the migration process (Kriegstein & Noctor, 2004; LoTurco & Bai, 2006). Cells become multipolar when they exit the VZ and switch to a bipolar shape when they locate in the IZ. By examining the morphology of the α5-shRNA R1-containing cells that accumulated in the premigratory zone, we found that many of them displayed a multipolar morphology, though abnormal, compared with the numerous bipolar cells transfected with control shRNA (Fig. 3B). Many of the α5-shRNA R1-containing multipolar cells presented a very irregular shape with several processes, as illustrated at high magnification in Fig. 3B.

**FIG. 4:**
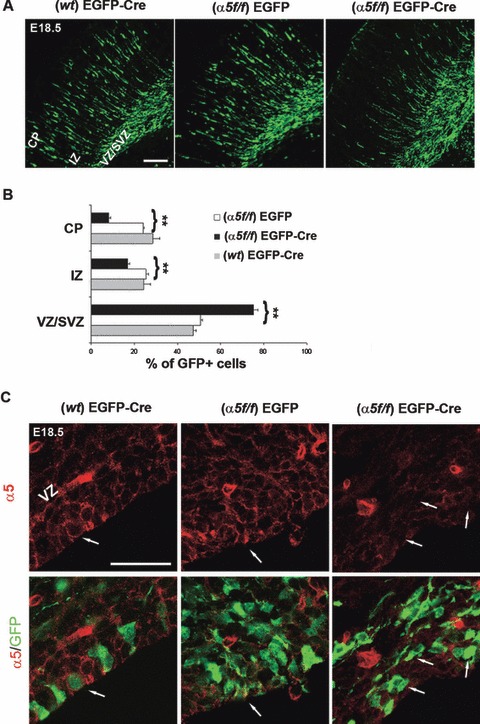
Impaired radial migration after deletion of the *α5* integrin gene. (A) Representative coronal sections of an E18.5 wild-type (*wt*) or floxed α5 (α5^*f/f*^) mouse brain transfected at E15.5 with EGFP or EGFP + Cre expression plasmids. The expression of Cre recombinase in α5^*f/f*^ mice inhibited radial migration. Scale bar, 100 μm. (B) Quantitative analyses of the distribution of EGFP-positive cells in the various cortical layers. Statistical differences were seen for each layer, comparing the distribution of EGFP-positive cells in the α5^*f/f*^ brain transfected with EGFP alone or EGFP + Cre plasmids. Asterisks indicate significant differences between the groups (**P < 0.01). (C) Reduction of α5 integrin expression in α5^*f/f*^ neural progenitors cells electroporated with EGFP + Cre plasmids. The white arrows indicate the same cells immunostained for α5 integrin (red) and GFP (green). Scale bar, 20 μm.

To corroborate the results obtained with selective suppression of α5 integrin in the neuroepithelium by RNAi, we used a totally different approach, which was to induce the deletion of the *α5* integrin gene by electroporating a vector encoding the Cre recombinase into cortical progenitor cells in the VZ of mice carrying an *α5 integrin-floxed* allele (*α5* ^*f/f*^, van der Flier *et al.*, unpublished data). To test for Cre activity of our Cre expression plasmid, we used *ROSA26* reporter mice (R26R), in which a stop cassette, flanked by loxP sites, inhibits *lacZ* gene expression (Soriano, 1999). We co-electroporated homozygous R26R/R26R embryos with EGFP and the pxCANCre plasmid at E15.5, and examined the Cre recombination activity based on the expression of β-galactosidase resulting from Cre-mediated deletion of the floxed stop cassette after 3 days. X-gal staining showed specific *lacZ* activity in the EGFP + Cre electroporated region, indicating that Cre-mediated recombination had occurred (Fig. S3). We then co-electroporated EGFP and pxCAN-Cre plasmids into *α5*^*f/f*^ mice (*n* = 3) and examined electroporated brains 3 days later. Migration defects were observed, comparable with those induced by α5 integrin silencing, with an accumulation of migrating neurons in the ventral region of the cortex (Fig. 4A). In contrast, in *α5*^*f/f*^ mice electroporated with only EGFP (*n* = 2) or in wild-type mice co-electroporated with EGFP and Cre vectors (*n* = 2), the transfected cortical neurons migrated normally away from the VZ/SVZ to reach the CP (Fig. 4A). Quantification of EGFP-positive cells in the VZ/SVZ, IZ and CP regions of electroporated brains confirmed that Cre electroporation in *α5* ^*f/f*^ mice impaired radial migration. Over 75% of EGFP+Cre+ *α5*^*f/f*^ cells remained in the VZ/SVZ, in contrast to 50% in control (EGFP only electroporations) (*P* < 0.01) (Fig. 4B). A fraction of EGFP+Cre+ *α5* ^*f/f*^ cells still initiated migration. By using markers of future layers V (Ctip2) (Leid *et al.*, 2004) or II/IV (Cux/Cdp) (Nieto *et al.*, 2004), a defect in migration was still present in these cells. As illustrated in Fig. S4, while control EGFP-*α5*^*f/f*^ cells had reached and passed Ctip2 or Cux/Cdp-positive layers at E18.5, this was not the case for EGFP+Cre+ *α5*^*f/f*^ cells, which were markedly delayed.

By staining coronal sections from *α5*^*f/f*^ brains co-electroporated with EGFP + Cre vectors with an α5 integrin antibody, we were able to see a reduction of the α5 signal in EGFP-positive cells accumulated in the VZ/SVZ, indicating that the impairment of migration is indeed occurring in cells in which Cre recombinase had induced *α5* integrin gene deletion (Fig. 4C).

As observed after electroporation of brains with siRNA R1 and R7, Cre-mediated deletion of α5 integrin gene in the cortical neural progenitors did not reveal gross morphological abnormalities in the glial processes or in the formation of glial endfeet (Fig. S5). In addition, morphological defects similar to those seen in the α5-shRNA-R1 neurons were detected in the EGFP+Cre+ *α5* ^*f/f*^ migrating neurons (see multipolar cells with irregular shapes carrying several processes in Figs S4 and S5).

**FIG. 5:**
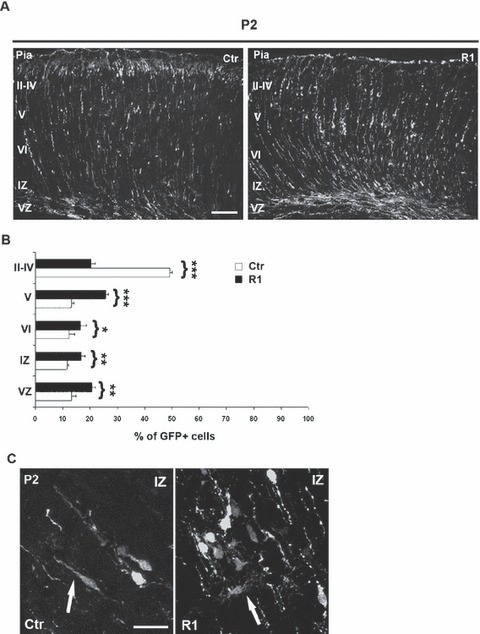
Effect of α5-shRNA on cerebral cortex laminar structure. (A) Representative coronal sections of embryonic murine neocortex 6 days (P2) following electroporation of Ctr and R1 constructs at E15.5. Transfection of R1 induces cortical lamination defects. Scale bar, 100 μm. (B) Quantitative analyses of the distribution of GFP-positive cells in the various cortical layers. Ctip-2 (red) labels layer V. Statistical differences were seen for each layer, comparing the distribution of GFP-positive cells between Ctr and R1 constructs. Asterisks indicate significant differences between the groups (***P < 0.001; **P < 0.005; *P < 0.02). (C) Morphology of neurons in IZ transfected with Ctr and R1 α5 shRNAs. Cells expressing shRNAs against α5 integrin showed atypical morphology compared with control transfected cells (white arrows). Scale bar, 50 μm.

As mentioned, neuronal progenitors transfected at E15.5 should primarily produce neurons in postnatal layers II–IV (Langevin *et al.*, 2007). To investigate whether down-regulation of α5 integrin had an effect on formation of cortical layers II–IV, we performed *in utero* electroporation of shRNA R1 at E15.5 and examined the positions of transfected cells 6 days later (P2), a stage when targeted neurons have completed radial migration (Creppe *et al.*, 2009). A marked migration delay was observed in R1 electroporated brains compared with controls (Fig. 5A). In control transfected brains (*n* = 3) 49.3 ± 0.8% of cGFP-positive cells reached layers II–IV. By contrast, in R1 electroporated brains (*n* = 3) only 20.3 ± 1.6% of α5-shRNA-expressing neurons were in layers II–IV (*P* < 0.001), whereas 58.9 ± 5.3% were located in cortical layers V and VI and in the IZ (Fig. 5B). Thus, the migration defects observed after 3 days still persist in the R1-transfected neurons after 6 days. In addition, in some regions along the cerebral cortex the α5-shRNA-expressing neurons formed clusters and showed morphological abnormalities with formation of multiple neuronal protrusions (Fig. 5C). Similar morphological defects were already observed 3 days after electroporation in R1-transfected cells that accumulated in the ventricular region (Fig. 3B), and in the EGFP+Cre+ *α5*^*f/f*^ migrating neurons (Figs S4 and S5), suggesting that α5 integrin could play a role in regulating neuronal polarity.

**FIG. 6:**
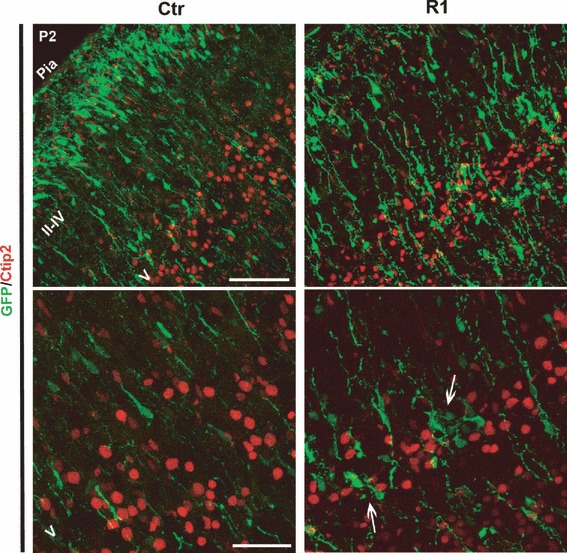
Reduced expression of α5 integrin has no effect on neuronal differentiation. Electroporated brains were examined for the localization of the cortical marker Ctip2. In control and R1-transfected brains, neurons from layer V showed normal expression of Ctip2. Bottom panels are magnified views of layer V. The white arrows indicate neurons blocked in layer V but not expressing Ctip2. Scale bars: top, 50 μm; bottom, 20 μm.

Interestingly, many α5-shRNA-expressing neurons were located in layer V (25.8 ± 0.8%). This ectopic positioning could be due to defects in neuronal differentiation, as interactions of cells with the ECM are known to be critical for the establishment and maintenance of cell fate (Czyz & Wobus, 2001). To test this hypothesis, we performed immunostaining for Ctip2, a specific marker for layer V (Leid *et al.*, 2004). As illustrated in Fig. 6, the α5-shRNA-expressing neurons accumulated in layer V did not express the Ctip2 transcription factor, suggesting that they were not typical deep layer V neurons (Fig. 6). In agreement with this result, staining at early stages of R1-electroporated brains with an antibody against βIII tubulin (Tuj1) did not reveal obvious differences in the expression of this neuronal marker or ectopic labeling in the VZ, as compared with control brains (G. Marchetti *et al.*, unpublished observations). Together, our results indicate that the knockdown of α5 integrin in neural progenitors at E15.5 induces a delay in radial migration in the cerebral cortex associated with lamination defects at the level of cortical layers II–IV.

## Discussion

This study provides evidence that down-regulation of the α5β1 integrin in the developing murine cerebral cortex impairs neuronal migration. We used an RNAi approach associated with *in utero* electroporation to reduce the expression level of α5 integrin in the neural progenitor cells in the VZ of E15.5 neocortex. By using two different shRNA sequences targeted against the *α5 integrin* gene, neuronal migration was inhibited. Both shRNA constructs reduced α5 integrin protein levels, whereas control shRNA constructs did not. We conclude that the impairment of neuronal migration was specifically due to the inhibition of α5 integrin expression. To exclude the possibility that the effect of α5 integrin shRNA on neuronal migration was dependent on potential off-target effects, we also used a different approach, which was to induce deletion of the *α5 integrin* gene in the cortical neural progenitors by Cre-mediated recombination. Migration defects were also observed with this approach. Therefore, by using two different approaches to inhibit expression of α5 integrin in the VZ of the developing neocortex, we were able to show that α5 integrin is required for the proper migration of cortical neurons. In agreement, it has been shown that tectal progenitors transfected with an antisense integrin β1 RNA by retroviral infection failed to migrate into the chicken tectal plate (Galileo *et al.*, 1992).

The majority of the transfected cells that accumulated in the ventricular region of brains electroporated with R1 showed a multipolar morphology. Morphological defects were also observed in neurons in which the *α5 integrin* gene had been deleted. It has been proposed that several substages of multipolar migrating neurons exist, which differ by their migration rate, and may correspond to different stages of migration (LoTurco & Bai, 2006). All of them switch to a bipolar shape when they exit the SVZ. This morphological transition requires a reorganization of the cytoskeleton. How these morphological changes are regulated remains to be elucidated. Molecules such as LIS1, doublecortin (DCX), Cdk5 and filamin A are necessary at different steps of this transition (LoTurco & Bai, 2006). Nagano *et al.* (2004) reported that FLNa, an actin-binding protein, acted on neuronal polarity and motility during mouse corticogenesis. They demonstrated that filamin A is involved in the multipolar-to-bipolar transition in migrating neocortical neurons. Indeed, filamin A reduction produces early arrest of multipolar cells in the SVZ similarly to integrin α5 knockdown while DCX RNAi induces ‘late’ arrest (LoTurco & Bai, 2006). As it has been shown that filamin A interacts with different integrin receptors and alterations of these interactions could affect cell migration and adhesion (Calderwood *et al.*, 2001), it is possible that α5β1 integrin and FLNa play cooperative roles in neuronal migration and interact during cerebral cortex formation to regulate the transition from multipolar to bipolar morphology.

Several integrin mutations result in radial glial defects at the basement membrane, producing marginal zone heterotopia. Belvindrah *et al.* (2007) found that, in mice with a specific deletion of the *β1 integrin* (*Itgb1*) gene in the post-mitotic neurons, neocortical development occurred normally. They concluded that β1 integrins are essential in radial glia but not in neurons for the migration and proper development of the cerebral cortex. In contrast, in our studies, down-regulation of α5 integrin in the cortical neural precursors affects neuronal migration. These discrepancies could be due to acute knockdown of α5 integrin bypassing compensatory mechanisms that are implemented with genomic disruption. Indeed, acute loss-of-function approaches revealed functions in the case of the *doublecortin* or *connexin* genes (Bai *et al.*, 2003; Elias *et al.*, 2007). This down-regulation occurs in neural progenitors and could thus affect expression of α5 integrin in either or both neuroblasts and radial glia. However, under our conditions, radial glial fibers did not show marked defects, suggesting that α5 integrin does not play an essential role in glial morphology, consistent with the fact that we did not detect a clear α5 immunoreactivity in radial glial fibers. It should be kept in mind that at the stage that we performed *in utero* electroporation (E15.5), the glial scaffold could be already established. Indeed, electroporation at E11 and E13 of vectors which allow the expression of Cre recombinase into embryonic brains of β1 integrin conditional mutant mice leads to stunted glial processes and lack of glial endfeet (Radakovits *et al.*, 2009). This phenotype is more severe than that observed in α6 integrin total knock-out mice, suggesting that other α subunits might be implicated (Georges-Labouesse *et al.*, 1998). To clarify if α5β1 contributes to early stages of organization of glial fibers and formation of glial endfeet, it would be necessary to inhibit its expression at much earlier stages. In addition, although multipolar cells may not be always in contact with the gliaI scaffold, further analyses will be required to elucidate if subtle alterations of neuron/glia interactions contribute to the migration defects observed.

In contrast to experiments such as the knock-down of the elongator complex, in which delay in migration is temporary and no longer visible at P2 (Creppe *et al.*, 2009), we observed that the alterations of distribution of knocked-down α5 neurons persisted 6 days after electroporation at P2. Indeed, under control conditions, the majority of electroporated cells reached layer II–IV, while cells expressing α5-shRNA R1 were located in layer V, which could have resulted from their altered fate or from defects in neuronal migration. Recent studies have revealed that regulation of integrin-mediated cell–matrix adhesions plays a role in differentiation of neuronal cells (Hajdo-Milasinovic *et al.*, 2007). Interestingly, α5-shRNA-R1-expressing neurons accumulated in the cortical layer V were not positive for the layer V marker Ctip2. Therefore, reduction in α5 integrin expression did not affect differentiation of neuronal progenitors. Moreover, some α5-shRNA-R1-expressing neurons showed an atypical morphology and an altered neuron polarity with misoriented neurites. These findings confirmed that the down-regulation of α5 integrin impairs radial migration, acting on neuronal morphology regulation.

In conclusion, our findings demonstrate that in early steps of neuronal migration in the cerebral cortex, the α5β1 fibronectin receptor promotes migration away from the VZ/SVZ, and may also have a role in the regulation of neuronal morphology.

## Abbreviations

BrdU: bromodeoxyuridine
Cdp: CCAAT-displacement protein
CP: cortical plate
Ctip2: chicken ovalbumin upstream promoter transcription factor-interacting protein 2
DAPI: 4′,6-diamidino-2-phenylindole
ECM: extracellular matrix
EGFP: enhanced green fluorescent protein
FN: fibronectin
GAPDH: glyceraldehyde 3-phosphate dehydrogenase
GFP: green fluorescent protein
IZ: intermediate zone
LN: laminin
MZ: marginal zone
PBS: phosphate-buffered saline
pH3: phospho-histone H3
R26R: *ROSA26* reporter mice
shRNA: short hairpin RNA
SVZ: subventricular zone
VZ: ventricular zone
α5^*f/f*^: alpha5 integrin floxed mice

## Supporting Information

Fig. S1. Expression of α5, α6 and β1 integrinsubunits in the developing cerebral cortex.

Fig. S2. Effects of α5 integrin down-regulation on cellproliferation and cell death of neural progenitors.

Fig. S3. Cre recombinase electroporation mediates efficient excision in the developing cerebral cortex.

Fig. S4. Impairment of cortical layering at E18.5 after deletion of the *α5* integrin gene.

Fig. S5. Effects of *α5* integrin gene deletion on radial glial scaffold and neuronal morphology.

As a service to our authors and readers, this journal provides supporting information supplied by the authors. Such materials are peer-reviewed and may be re-organized for online delivery, but are not copy-edited or typeset by Wiley-Blackwell. Technical support issues arising from supporting information (other than missing files) should be addressed to the authors.
