# Supplementary material for: Integrin α5β1 is necessary for regulation of radial migration of cortical neurons during mouse brain development
Source: Eur J Neurosci. 2010 Feb;31(3):399–409. doi: 10.1111/j.1460-9568.2009.07072.x (PMC3460545; doi:10.1111/j.1460-9568.2009.07072.x)
Supplement: Supplementary file 1 [file ejn0031-0399-SD1.doc]

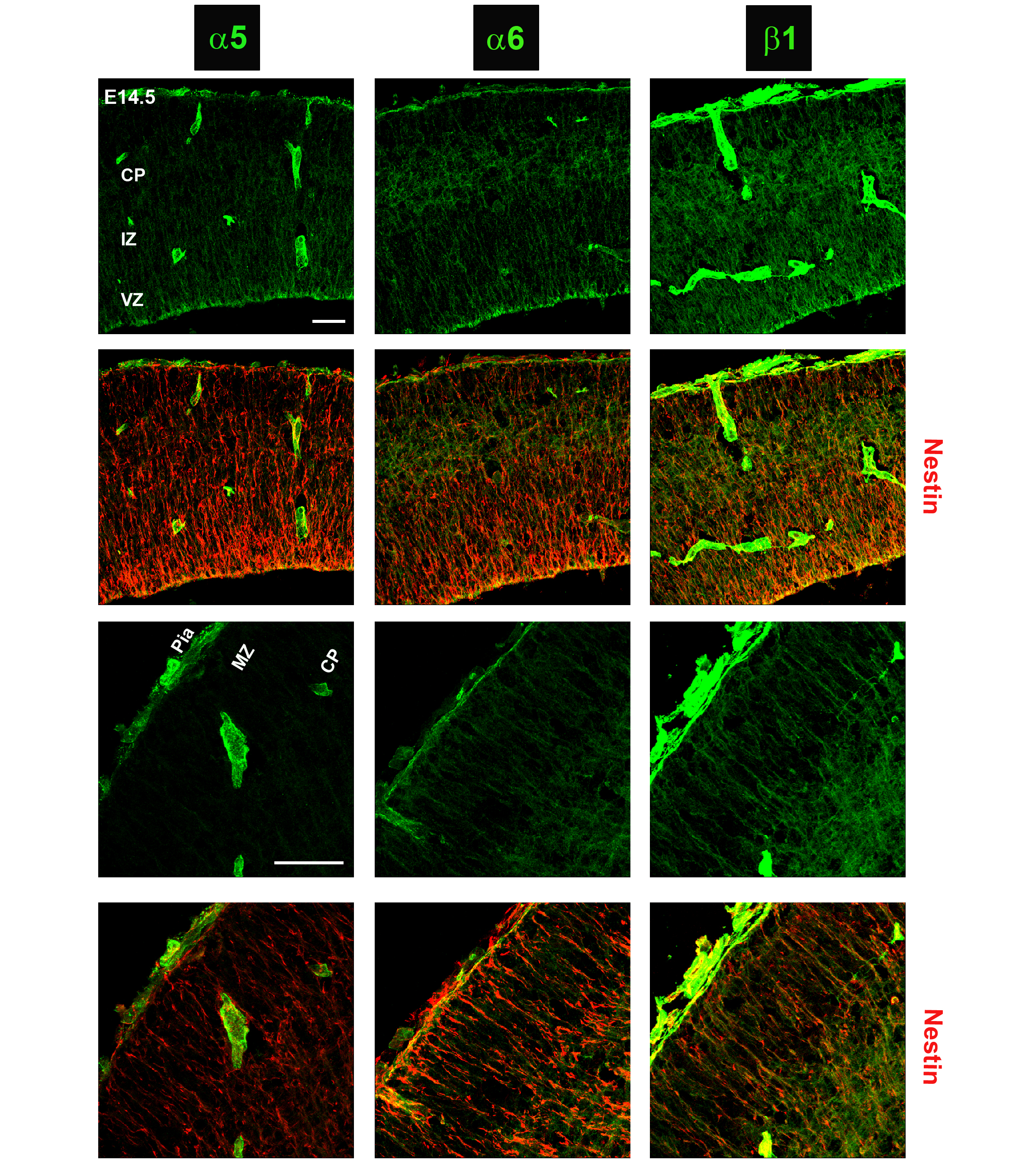


**Fig. S1. Expression of α5, α6 and β1 integrin subunits in the developing cerebral cortex.**

Coronal sections of E14.5 mouse brains were stained with antibodies directed against α5, α6 and β1 integrin chains (green) and with anti-Nestin antibody (red). All three integrin subunits were expressed in the VZ while α6 and β1 integrin were also localized in the CP. Scale bar, 100 μm. Bottom panels are magnified views of the MZ. In this cortical zone, the α6 and β1 integrins are present in the glial radial fibers whereas α5 integrin subunit expression is visible only in the blood vessels and not in glial fibers. Scale bar, 50 μm.
