# Supplementary material for: Integrin α5β1 is necessary for regulation of radial migration of cortical neurons during mouse brain development
Source: Eur J Neurosci. 2010 Feb;31(3):399–409. doi: 10.1111/j.1460-9568.2009.07072.x (PMC3460545; doi:10.1111/j.1460-9568.2009.07072.x)
Supplement: Supplementary file 2 [file ejn0031-0399-SD2.doc]

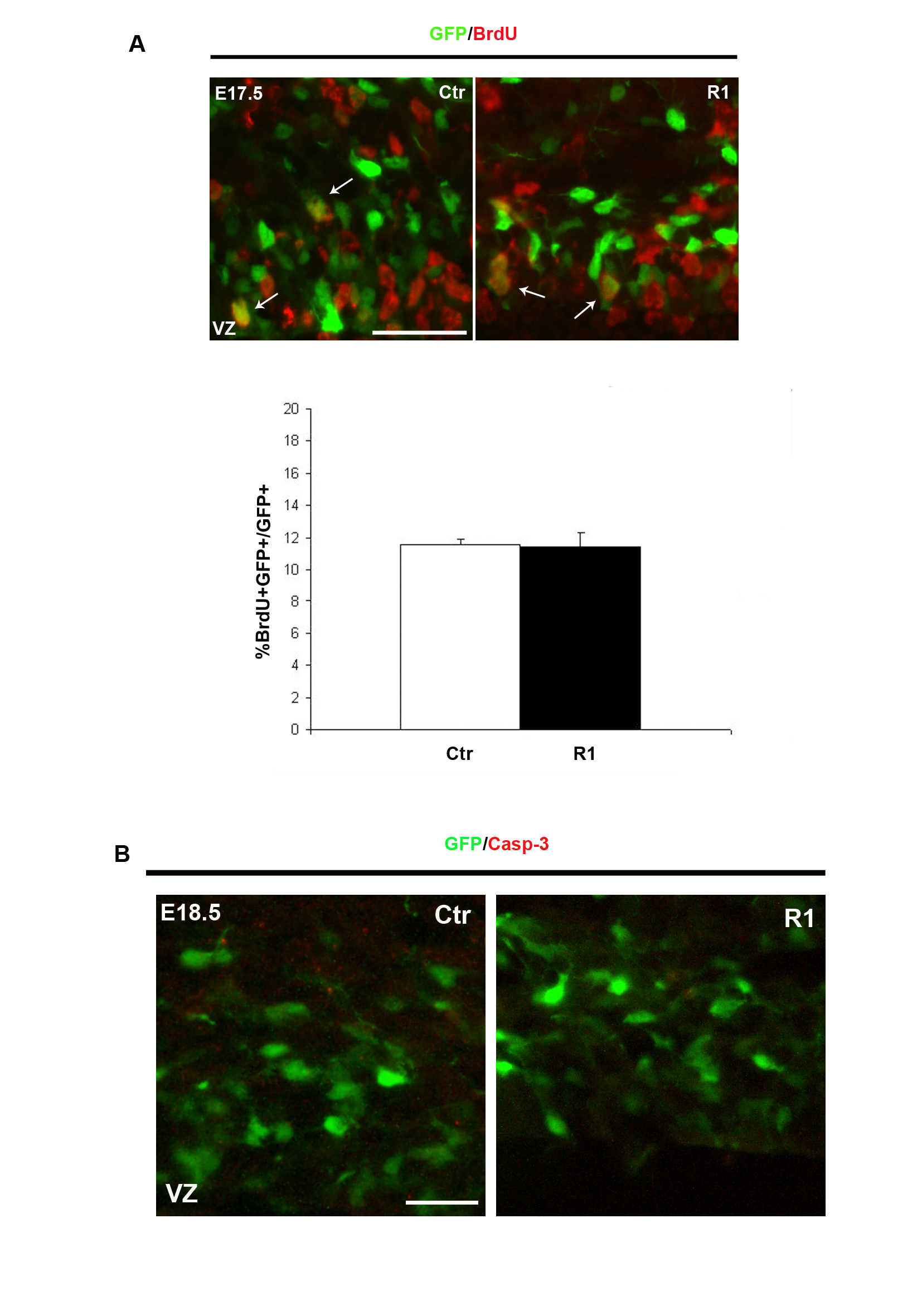


**Fig. S2. Effects of α5 integrin dowregulation on cell proliferation and cell death of neural progenitors**.

(A) Control or R1 α5-shRNA constructs were electroporated into E15.5 embryonic brains. Mice were injected with BrdU at E17.5 and sacrificed 2 hr later. Coronal sections of electroporated embryonic brain were costained with anti-GFP (green) and anti-BrdU (red). Arrows indicate GFP and BrdU double-positive cells. Histogram depicting the percentage of BrdU and GFP double-positive cells to total GFP-positive cells in VZ. Scale bar, 20 μm. (B) Coronal sections from E18.5 electroporated brain with Ctr or α5-shRNA constructs and stained with anti-activated caspase-3 (red) and GFP (green). Scale bar, 50 μm.
