# Supplementary material for: Integrin α5β1 is necessary for regulation of radial migration of cortical neurons during mouse brain development
Source: Eur J Neurosci. 2010 Feb;31(3):399–409. doi: 10.1111/j.1460-9568.2009.07072.x (PMC3460545; doi:10.1111/j.1460-9568.2009.07072.x)
Supplement: Supplementary file 3 [file ejn0031-0399-SD3.doc]

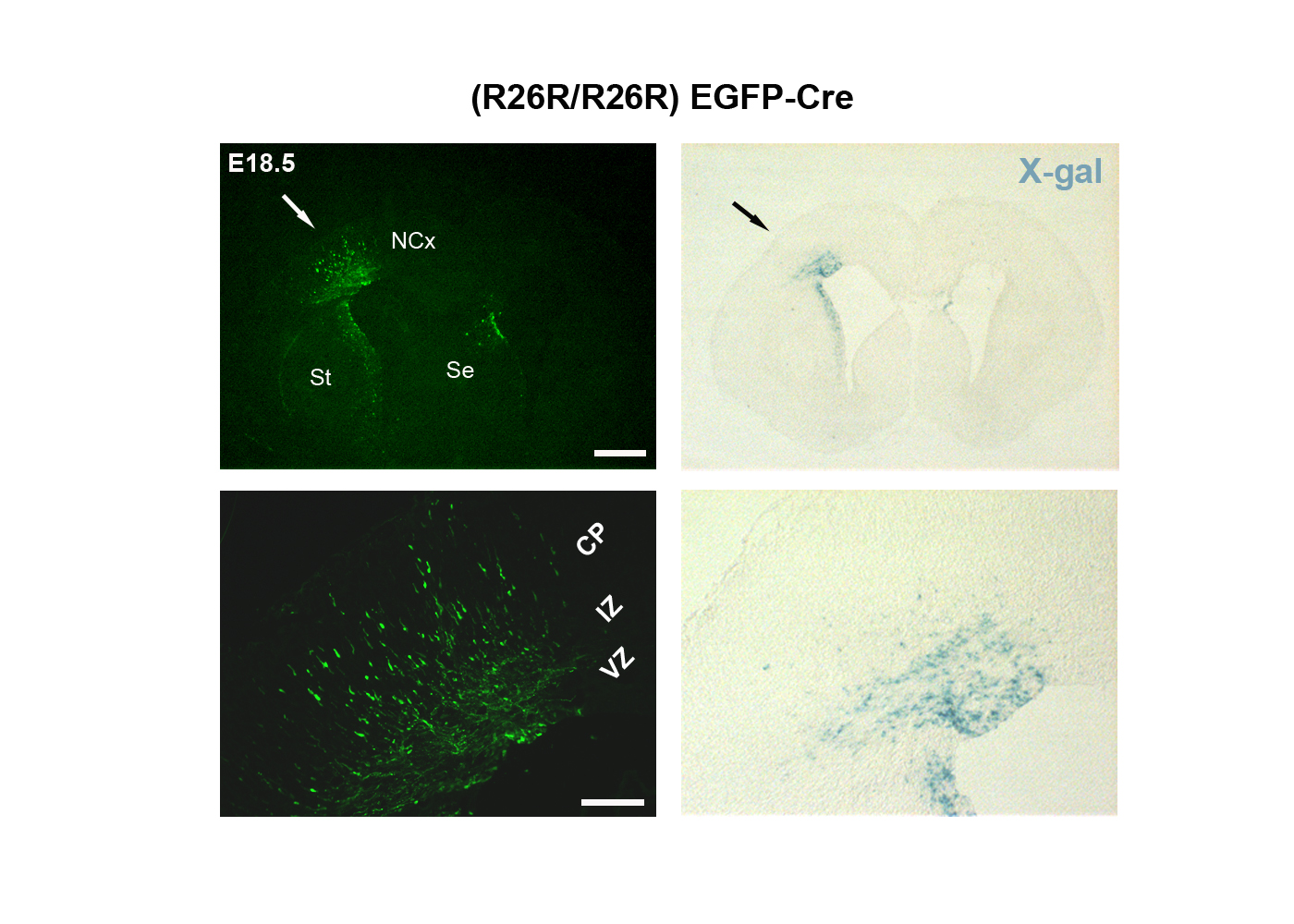


**Fig. S3. Cre recombinase electroporation mediate efficient excision in the developing cerebral cortex.**

(A) Coronal sections of an E18.5 R26R/R26R brain coelectroporated with EGFP + Cre plasmids. The arrows indicate the electroporated telencephalic hemisphere. In the left panel, the GFP staining allows visualization of the transfected cells. In the corresponding region of the right panel, β-galactosidase staining showed *lacZ* expression upon Cre recombination. Scale bar, 500 μm. Bottom panels are magnified views of the electroporated region. Scale bar, 100 μm. NCx, neocortex ; St, striatum ; Se, septum.
