# Supplementary material for: Integrin α5β1 is necessary for regulation of radial migration of cortical neurons during mouse brain development
Source: Eur J Neurosci. 2010 Feb;31(3):399–409. doi: 10.1111/j.1460-9568.2009.07072.x (PMC3460545; doi:10.1111/j.1460-9568.2009.07072.x)
Supplement: Supplementary file 4 [file ejn0031-0399-SD4.doc]

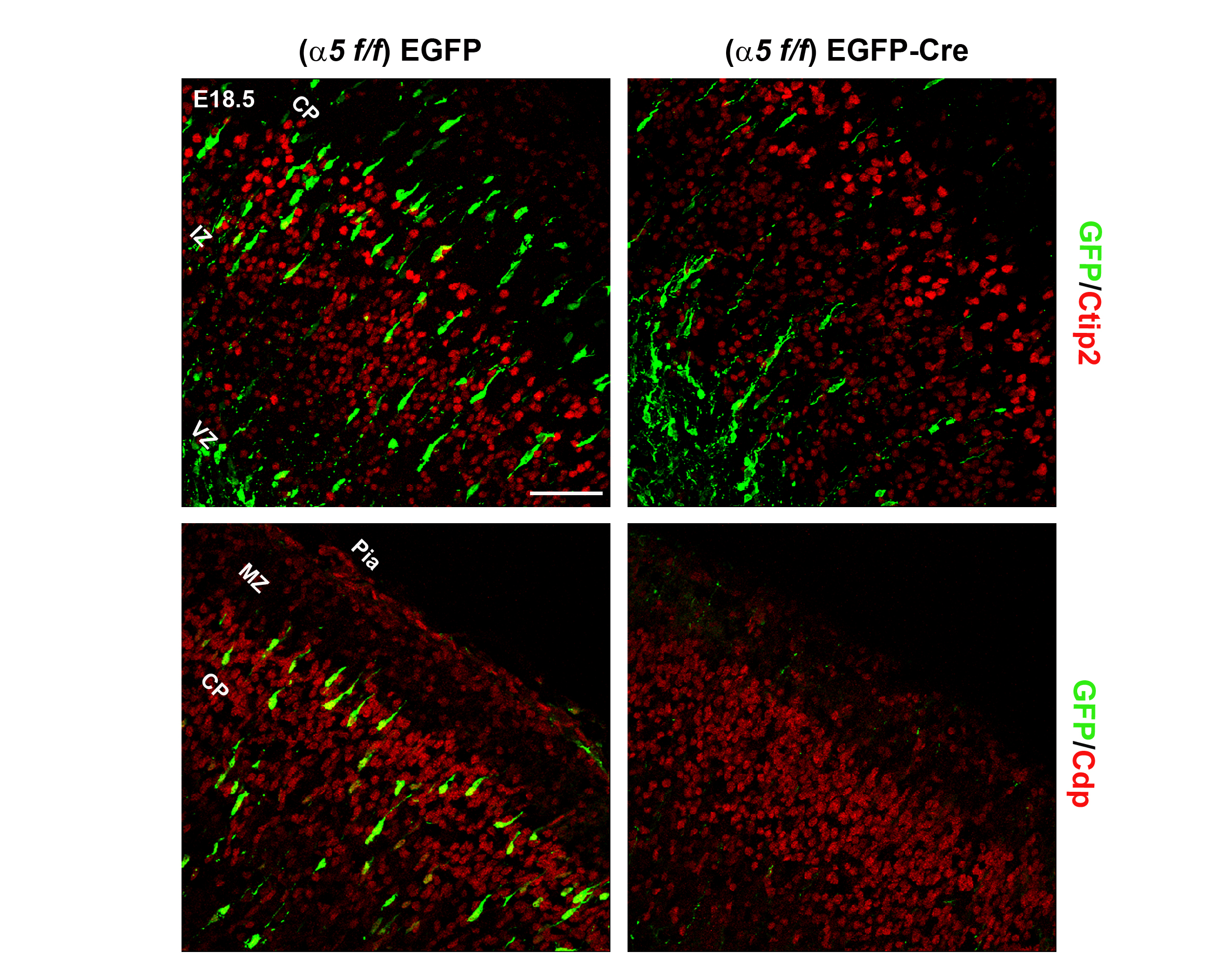


**Fig. S4. Impairment of cortical layering eat E18.5 after deletion of the *α5* integrin gene.**

Coronal sections of an E18.5 floxed α5 (α5*^f/f^*) mouse brain transfected at E15.5 with EGFP or EGFP + Cre expression plasmids were costained for GFP (green) and the cortical prospective layer V marker Ctip2 or layer II/IV marker Cdp (red). In the control electroporated brains, migrating GFP-positive cells are seen that will position into the cortical layer II-IV. In contrast, the Cre recombinase expressing (GFP-positive) α5*^f/f^* neural cells cells appear very delayed and misplaced. Many cells show an abnormal morphology. Scale bar, 50 μm.
