# Supplementary material for: Integrin α5β1 is necessary for regulation of radial migration of cortical neurons during mouse brain development
Source: Eur J Neurosci. 2010 Feb;31(3):399–409. doi: 10.1111/j.1460-9568.2009.07072.x (PMC3460545; doi:10.1111/j.1460-9568.2009.07072.x)
Supplement: Supplementary file 5 [file ejn0031-0399-SD5.doc]

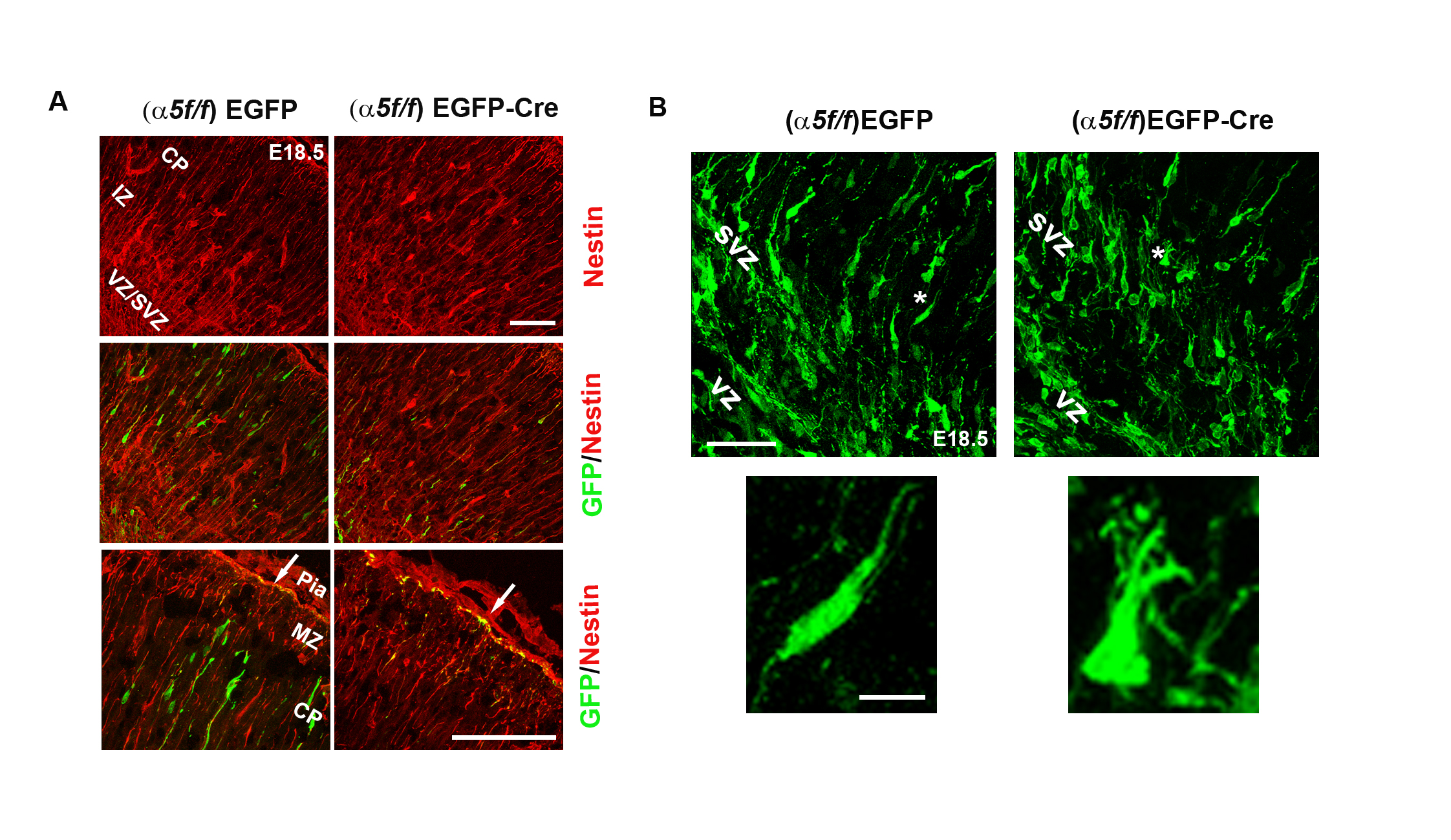


**Fig. S5. Effects of *α5* integrin gene deletion on radial glial scaffold and neuronal morphology**

(A) Coronal sections of E18.5 floxed α5 (α5*^f/f^*) mouse brains, electroporated at E15.5 with EGFP or EGFP + Cre expression plasmids, were stained with anti-Nestin (red) and GFP (green) antibodies. Scale bar, 100 μm. Bottom panels are magnified views of the MZ. The white arrows indicate basal endfeet of radial fibers attached to the pia. The expression of Cre recombinase in α5*^f/f^* mice does not seem to induce gross abnormalities in the glial scaffold organization or glial endfeet formation. Scale bar, 50 μm.

(B) Morphology of VZ/SVZ α5*^f/f^* neurons transfected with EGFP or EGFP + Cre expression plasmids. The α5*^f/f^* electroporated cells with EGFP showed a normal bipolar morphology in the premigratory region (white asterisk, high magnification). In contrast, α5*^f/f^* cells expressing also Cre recombinase are arrested within the VZ/SVZ with an abnormal shape (white asterisk, high magnification). Scale bar, top: 50 μm, bottom: 10 μm.
